# A challenging playing field: Identifying the endogenous impediments to annulus fibrosus repair

**DOI:** 10.1002/jsp2.1133

**Published:** 2021-02-11

**Authors:** Ana P. Peredo, Sarah E. Gullbrand, Robert L. Mauck, Harvey E. Smith

**Affiliations:** ^1^ Department of Bioengineering School of Engineering and Applied Science, University of Pennsylvania Philadelphia Pennsylvania USA; ^2^ McKay Orthopaedic Research Laboratory, Department of Orthopaedic Surgery Perelman School of Medicine, University of Pennsylvania Philadelphia Pennsylvania USA; ^3^ Translational Musculoskeletal Research Center Corporal Michael J. Crescenz Veterans Affairs Medical Center Philadelphia Pennsylvania USA

**Keywords:** annulus fibrosus, annulus fibrosus repair, disc herniation, discectomy, endogenous repair

## Abstract

Intervertebral disc (IVD) herniations, caused by annulus fibrosus (AF) tears that enable disc tissue extrusion beyond the disc space, are very prevalent, especially among adults in the third to fifth decade of life. Symptomatic herniations, in which the extruded tissue compresses surrounding nerves, are characterized by back pain, numbness, and tingling and can cause extreme physical disability. Patients whose symptoms persist after nonoperative intervention may undergo surgical removal of the herniated tissue via microdiscectomy surgery. The AF, however, which has a poor endogenous healing ability, is left unrepaired increasing the risk for re‐herniation and pre‐disposing the IVD to degenerative disc disease. The lack of understanding of the mechanisms involved in native AF repair limits the design of repair systems that overcome the impediments to successful AF restoration. Moreover, the complexity of the AF structure and the challenging anatomy of the repair environment represents a significant challenge for the design of new repair devices. While progress has been made towards the development of an effective AF repair technique, these methods have yet to demonstrate long‐term repair and recovery of IVD biomechanics. In this review, the limitations of endogenous AF healing are discussed and key cellular events and factors involved are highlighted to identify potential therapeutic targets that can be integrated into AF repair methods. Clinical repair strategies and their limitations are described to further guide the design of repair approaches that effectively restore native tissue structure and function.

## 1 INTRODUCTION

The intervertebral disc (IVD) is essential for motion and the transmission of forces across the spine. IVDs connect adjacent vertebra in the spinal column, providing flexibility and multi‐axial range‐of‐motion. The IVD achieves its function by having a composite structure comprised of three elements that work in concert to withstand the complex loads that arise during body motion.^1^, ^2^ The tissue consists of a central hydrated, proteoglycan‐rich nucleus pulposus (NP) surrounded circumferentially by a fibrocartilaginous annulus fibrosus (AF) and by thin cartilaginous endplates (CEPs) at the top and bottom edges.^3^, ^4^ Once the IVD is loaded, the NP is compressed and rapidly pressurized, causing it to expand outward radially into the AF. The AF plays an indispensable role in the load‐bearing process by restraining the NP circumferentially and resisting extension and torsion, which result in a combination of compressive, tensile, and shear forces.^2^, ^5^, ^6^, ^7^, ^8^

**FIGURE 1:**
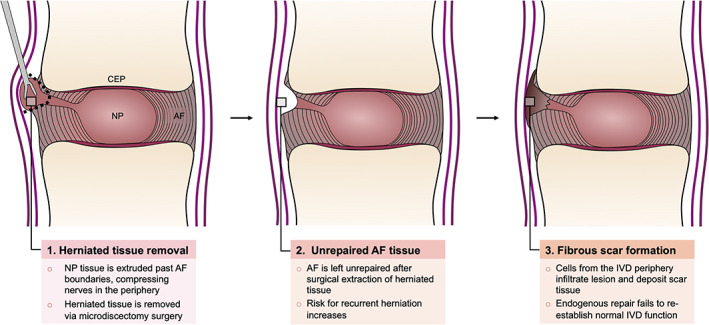
**Conventional surgical approach for symptomatic IVD herniations** **.** AF injuries enable the NP to be extruded out of the IVD space. The herniated tissue often compresses adjacent nerves, causing significant pain and disability. Patients whose symptoms remain unresolved with nonsurgical approaches may undergo microdiscectomy surgery through which the herniated tissue is excised. The AF, however, is left unrepaired, which does not re‐establish IVD function and increases the risk for recurrent disc herniation. AF, annulus fibrosus; IVD, Intervertebral disc; CEP, cartilaginous endplates

Due to the central load‐bearing role and challenging mechanical demands of the AF, its structure often fails, resulting in herniation of the IVD. Disc herniations affect 2 to 3% of the world population and pose a significant socioeconomic burden.^9^, ^10^ Injuries to the AF arise from acute trauma or result from progressive degeneration of the AF. When the AF fails, the soft, central NP tissue is extruded through the lesion, often impinging adjacent spinal nerves and causing debilitating pain.^4^ These injuries compromise the integrity of the IVD composite structure, which leads to impaired mechanical function, inflammation, and exacerbated tissue degeneration.^11^, ^12^ Patients with persistent symptoms that are unresolved with nonoperative treatment may undergo microdiscectomy surgery, during which herniated NP and AF tissue is removed to relieve nerve impingement and pain.^13^ The AF, however, which has a low endogenous healing capacity, does not regenerate and fails to restore its native structure and composition. This increases the risk for re‐herniation after surgical intervention and pre‐disposes the IVD to degenerative disc disease (Figure 1).^14^

While methods for NP regeneration, ranging from molecular supplements to biomaterial‐guided repair, have been extensively pursued, AF repair strategies remain largely underdeveloped. This is due, in part, to an incomplete characterization of the endogenous molecular and cellular mechanisms involved in AF healing. Moreover, the lack of consensus on the phenotypic markers that are specific to AF cells impedes the advancement of cellular differentiation strategies for supplementation of AF lesions. There are currently no FDA‐approved medical devices that repair the AF postherniation. While a barrier device that is intended to obstruct the AF lesion postdiscectomy exists, this method does not promote biologic repair of the AF and consequently fails to restore native tissue mechanics and function. To address the need for new AF repair techniques, several groups have developed acellular and cellular delivery approaches to supplement the repair environment at the site of injury.^8^, ^15^, ^16^, ^17^ However, these methods have yet to successfully provide long‐term repair and restoration of IVD biomechanics.^18^, ^19^, ^20^, ^21^, ^22^, ^23^, ^24^, ^25^, ^26^, ^27^ This emphasizes the great need for the development of effective AF repair strategies that recapitulate native tissue function and minimize the occurrence of recurrent herniation.

Achieving robust integration with the native tissue, providing mechanical stability, and recovering AF biomechanics through the implantation of a repair structure presents significant challenges. The hierarchical AF composition and microstructural organization makes tissue recovery a complex endeavor. Similarly, the challenges presented by the intricate spinal anatomy impose constraints on the design of repair approaches. Providing and improving regenerative processes for the AF may propel the innovation of solutions that will mitigate the incidence of recurrent herniations. In this review we highlight the limitations of endogenous AF repair and identify key cellular events and factors involved postherniation to identify potential therapeutic targets. Currently available methods for clinical AF repair and their limitations are discussed to develop effective strategies for the restoration of normal tissue structure and function.

## 2 THE ANNULUS FIBROSUS

### 2.1 Structure, organization, and biochemical composition

The AF is a vital constituent of the IVD that is tasked with resisting the complex loads that arise during body movement. The AF must also be quite resilient, as it needs to re‐establish the tissue's normal arrangement with repetitive cycles of loading. To meet these mechanical and structural demands, the AF possesses a highly‐refined structural organization with regional variations in organization, biochemical composition, and cellular phenotype, dependent on the local mechanical loading environment.

Overall, the AF is composed of 15 to 25 coaxial lamellae made up of co‐aligned collagen fibers enmeshed with proteoglycans. Each lamella is connected with an interlamellar matrix that is rich in elastin and proteoglycan aggregates.^2^ The anterior AF has more lamellar layers than the posterior AF, due to the irregular bean‐shape morphology and loading conditions of the IVD in the axial plane.^28^ The collagen fibers that make up each lamella are highly aligned at oblique orientations, increasing from ±28° from the horizontal axis in the outer AF to ±43° in the inner AF, in an alternating orientation between successive lamellar layers.^1^ In addition to the graded orientation of fibers from the inner to outer AF, the layer stiffness also varies radially, with the outer AF having 2 to 3 times the stiffness of the inner AF.^29^ This is due to differences in collagen content and type: the outer AF contains mainly type I collagen while the inner AF is a mix of type I and type II collagen.^29^, ^30^, ^31^ Differences in lamellar stiffness are accompanied by a graded distribution of glycosaminoglycans (GAGs), where GAG content increases from 3% to 8% per wet weight from the outer to the inner AF, resulting in higher water content and permeability in the inner region of the tissue.^32^, ^33^ Consequently, outer lamellar layers have higher tensile strength than inner layers, with equilibrium moduli of 136 MPa and 59 MPa, respectively.^17^ The inner AF is more similar in composition to the NP, and can therefore more effectively resist compressive loads. Conversely, the outer AF, which is stiffer in tension, more effectively resists the large circumferential stresses that arise from NP radial pressure and the cranial‐caudal stretch that occurs during IVD loading.^7^, ^29^, ^34^, ^35^

### 2.2 Cellular phenotype and heterogeneity

The specialized matrix that comprises the inner and outer AF is maintained by a sparse population of cells in the mature AF. During postnatal development, the AF transitions from a highly cellular structure to a tissue with low cellularity that is rich in extracellular matrix (ECM). At birth, the anterior and posterior AF contain ~33 800 cells/mm^3^ and ~ 43 200 cells/mm^3^, respectively, which decrease to ~1500 cells/mm^3^ and ~ 1700 cells/mm^3^ by developmental maturity.^36^, ^37^, ^38^ Other studies have reported the adult AF cell density to range between 3000 to 9000 cells/mm^3^.^37^, ^39^ During fetal development, blood vessels are apparent deep within the AF, yet the vessels recede during postnatal growth, leaving behind pathways that remain as lamellar cross bridges in the avascular adult AF.^28^ The adult AF tissue also contains regional variations in cell density between the inner and outer regions; the outer adult AF has approximately 2.5 times the cellularity of the inner AF.^40^ Coinciding with these inner‐to‐outer variations, the AF cells in these distinct tissue regions display contrasting cellular morphologies. Cells in the inner region have round cell bodies and are similar in their cytoskeletal arrangement to NP cells. Outer AF cells, on the other hand, display a fibroblast‐like, elongated shape and are oriented along the collagen fibers. These cells have protrusions perpendicular to the lamellae through the surrounding dense matrix as a means for cellular communication.^34^, ^41^

In addition to inner and outer AF cells, a smaller population of cells have been described in the AF that reside in specialized compartments. Cells that reside in the interlamellar space display an irregular cytoskeletal arrangement, possibly due to the interlamellar shear forces they experience.^42^, ^43^ The specific expression profile and biosynthetic performance of this subpopulation has yet to be investigated.^34^ Similarly, a small population of stem cell‐like cells residing in the AF have been identified; these cells exist in a specialized niche near the perichondrium‐ and endplate‐AF borders.^44^, ^45^ Although these cells were identified based on their expression of mesenchymal stromal cell and pluripotency markers, their origin, function, and phenotype are less understood and warrant further investigation.^46^, ^47^

Over the years, several studies have tried to elucidate the cell surface, transcriptomic, and biosynthetic markers specific to the AF phenotype. A majority of the cells that make up the early postnatal murine AF express Scleraxis (Scx), a common marker for fibrous tissues.^48^ Interestingly, expression of this factor decreases with tissue maturity.^48^, ^49^ Additional genes that have been implicated in the formation of the IVD and that localize to the AF are Pax1 and Pax9, that encode paired box transcription factors.^50^ At E12.5, both inner and outer AF cells express Pax1/Pax9 in developing mouse embryos. However, by E15.5, expression is restricted to the outer AF.^51^ Mature AF cells are recognized for their biosynthesis of collagen I and very low COL2A1‐to‐COL1A1 gene expression ratios, compared to cells in articular cartilage and the NP.^52^, ^53^, ^54^ Additional ECM proteins that have been suggested as discriminating markers for the AF cell fate include collagen V and elastin.^53^ Although extensive gene expression analyses have elucidated differences between AF cells and cells residing in the NP and CEP structures, there is no general consensus as to what cellular characteristics are required to categorize a cell as an AF cell.^52^, ^54^, ^55^, ^56^, ^57^ Additional investigation of the cellular lineage and transcriptional profiles of AF cells via single cell sequencing and cell trajectory reconstruction are required to identify and characterize the origin and diversity of the cell populations that reside in the AF.

## 3 ANNULUS FIBROSUS INJURY

### 3.1 Injury manifestations, diagnosis and patient demographics

**FIGURE 2:**
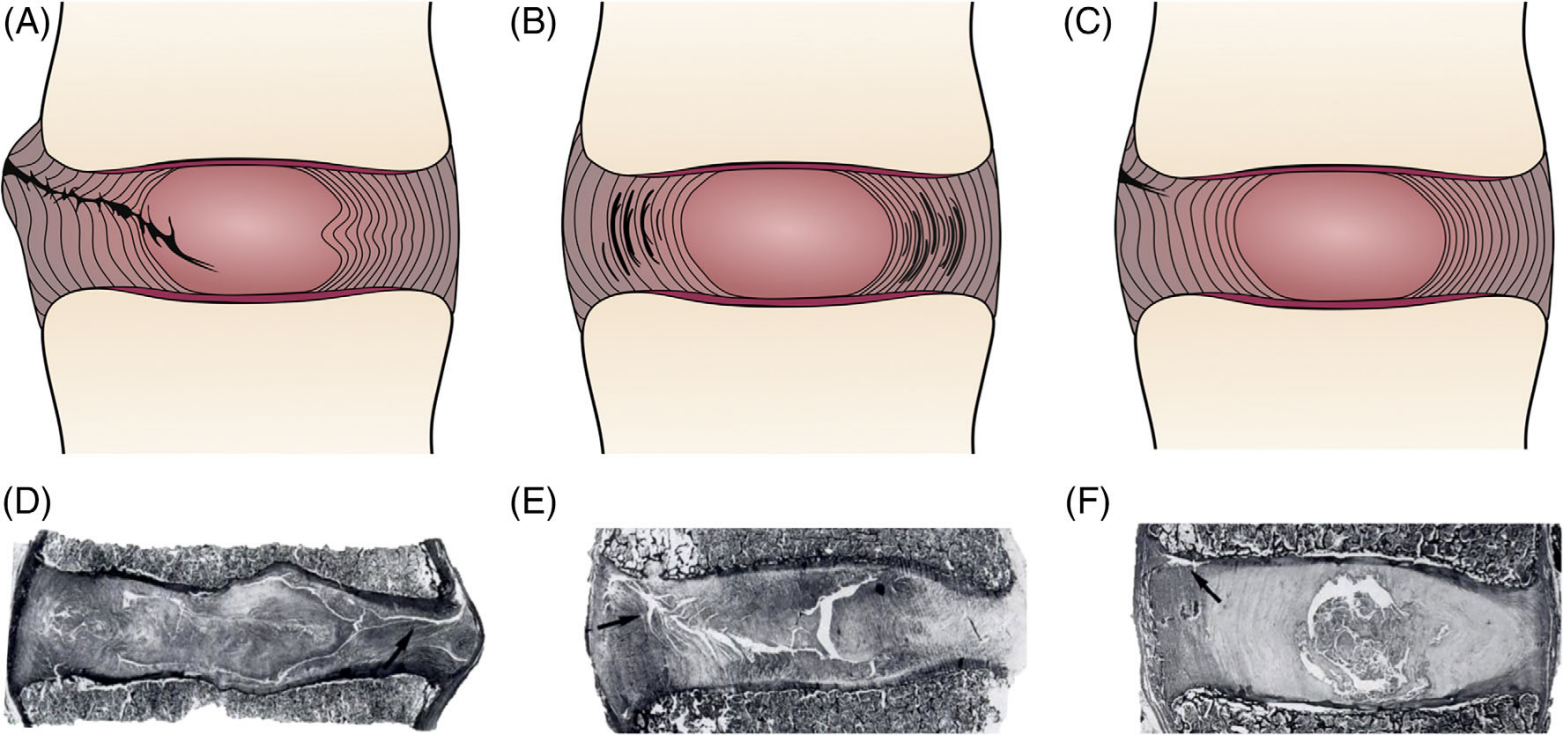
Types of annular tears commonly observed in human specimens. A, Radiating tears, commonly found in the posterior AF, traverse the AF structure and often reach the NP. B, Circumferential tears commonly occur along lamellar boundaries in outer or inner AF regions. C, Rim lesions are often found along the AF‐CEP boundary. D, L5‐S1 IVD of a 47‐year‐old woman with a radiating tear (arrow) extending towards AF periphery, producing infoldings of the inner AF layers. E, L4‐L5 IVD of a 50‐year‐old man with multiple circumferential tears extending towards a rim lesion (arrow). F, L2‐3 IVD of a 39‐year‐old woman with a rim lesion (arrow) at the anterior edge of the upper vertebra. (d‐f) Figures were republished with permission from The Journal of Bone and Joint Surgery. British Volume.64. AF, annulus fibrosus; CEP, cartilaginous endplates; IVD, Intervertebral disc

The extent to which disc tissue extends past the IVD boundary results in diverse manifestations of disc injury. As a result, the terms herniation, protrusion, and bulging have been used interchangeably, although they represent distinct pathologies and should not be confused. According to the North American Spine Society, the American Society of Spine Radiology, and the American Society of Neuroradiology, a disc herniation is a localized displacement of disc tissue beyond the limits of the IVD space.^58^ Herniations occur through AF discontinuities that enable the protrusion, extrusion, or sequestration of NP tissue past the AF boundary. A protrusion is defined if the greatest distance between the edges of the disc material beyond the disc boundaries is less than the distance of the base, in any plane. An extrusion is defined when the diameter of the disc material beyond the disc boundaries is greater than the diameter of the base, in any plane.^59^ Sequestrations, on the other hand, lack continuity between the herniated tissue and the IVD. Bulging discs occur when extension beyond the disc margin is 50% to 100% of the circumference and may be either symmetrical or asymmetrical.^59^ Acute tissue injury through excessive repetitive loading or a single overload of the tissue or from chronic degeneration of the IVD (characterized by dehydration of the NP and fraying of the AF) results in annular tears that give way to extrusion of the NP. Tears that are commonly observed in human specimens can be classified into three types: (a) radiating tears that traverse the AF structure, commonly found in the posterior AF where the AF is thinner and has fewer lamellar layers; (b) circumferential tears along lamellar boundaries in outer or inner AF regions; and (c) rim lesions that occur along the boundary between the AF and the CEP (Figure 2).^60^

The origin and nature of these lesions have been extensively investigated for many decades through the analysis of human cadaveric specimens and injury studies using small and large animal models. With aging, the IVD undergoes natural degeneration that predisposes the tissue to injury. Small clefts in the AF have been observed in patients as young as 3 years of age, and these steadily increase in number and severity with age.^61^, ^62^ This is accompanied by a gradual loss of the distinct boundary between the NP and inner AF, which may give way to the formation of fissures through the AF. Circumferential tears are thought to result from torsional stress and to precede IVD degeneration.^60^, ^63^ These are commonly identified in disc specimens from 10 to 30 year‐old humans.^62^, ^64^, ^65^ Rim lesions are common in lumbar IVDs and are thought to be the result of trauma, as they precede the onset of degenerative changes in the disc itself.^66^ Radial lesions have a low incidence in young discs and become progressively more frequent in the elderly.^62^ An examination of 300 cadaveric lumbar spines, however, led to the conclusion that these radiating injuries are the result of tearing rather than tissue degeneration, given the observed ingrowth of blood vessels around the outside margin of the clefts.^63^ These findings indicate that AF lesions not only occur as a result of aging and tissue degeneration, but also that healthy, younger individuals may present with AF lesions that precede degenerative changes in other IVD components.

IVD herniations have been extensively studied clinically. The Spine Patient Outcomes Research Trial investigated IVD herniations in a multi‐center prospective study that consisted of both randomized and observational cohorts.^67^ The incidence of disc herniation was reported to vary with populations and risk factors, between 5 to 20 cases/1000 adults with a predominance in males of an average age of 41 years.^68^ The highest prevalence of IVD herniations is observed in people aged 30 to 50 years, with approximately 95% of herniated discs found in the lower lumbar spine.^69^ Disc herniations above the lumbar spine are more common in people over 55 years of age.^69^ Lumbar disc herniations most commonly result from tears in the posterolateral AF, which can cause pain via mechanical compression of nerve roots or chemical irritation through an active inflammatory cascade.^70^ Interestingly, about 19% to 27% of disc herniations are asymptomatic. The lack of symptoms may be a consequence of the nature of the tear, location, and severity of the injury. Symptomatic lumbar disc herniations that do not resolve with initial appropriate nonoperative management are often managed surgically.^71^ While surgical intervention decompresses neural elements and removes the herniated component of the disc, there is no successful method to clinically repair the AF. While there are clinical strategies to suture or seal annular defects, these strategies do not biologically restore the annulus structure and biomechanics. Consequently, patients that have incurred disc herniations have a risk for recurrent herniation, with larger annular defects being associated with an increased risk of re‐herniation.^70^, ^72^

## 4 CLINICAL AF REPAIR

The clinical treatment of annular lesions is tailored to the relief of patient symptoms. Disc bulging or herniations can often result in back or leg pain, numbness, and tingling, among other symptoms, due to nerve compression and radiculopathy. When noninvasive approaches, such as physical therapy, fail to resolve patient symptoms, surgical removal of the herniated disc tissue is performed via open discectomy, endoscopic discectomy, or micro‐discectomy, depending on the location and severity of the injury.^73^, ^74^, ^75^ During the procedure, herniated tissue that has extruded beyond the AF boundaries is removed to decompress the symptomatic nerves. In general, discectomy is successful at alleviating pain and enabling patients to return to normal daily activities; however, this procedure does not repair the AF lesion and consequently, 10% to 30% of patients experience symptomatic recurrent herniation.^14^

Current surgical interventions for disc herniation also do not consider the long‐term effects of altering the IVD structure. When the herniated NP tissue is extracted, but not replaced, the reduction in NP volume hinders IVD pressurization and reduces disc height.^9^ Similarly, when herniated AF fragments are excised and the AF tear is not repaired during surgery, the AF cannot effectively restrain the NP during axial loading.^76^, ^77^ These physical changes alter the biomechanics of the disc and increase the risk for disc space collapse, spondylosis, abnormal facet loading, and disc degeneration.^9^, ^78^, ^79^

**FIGURE 3:**
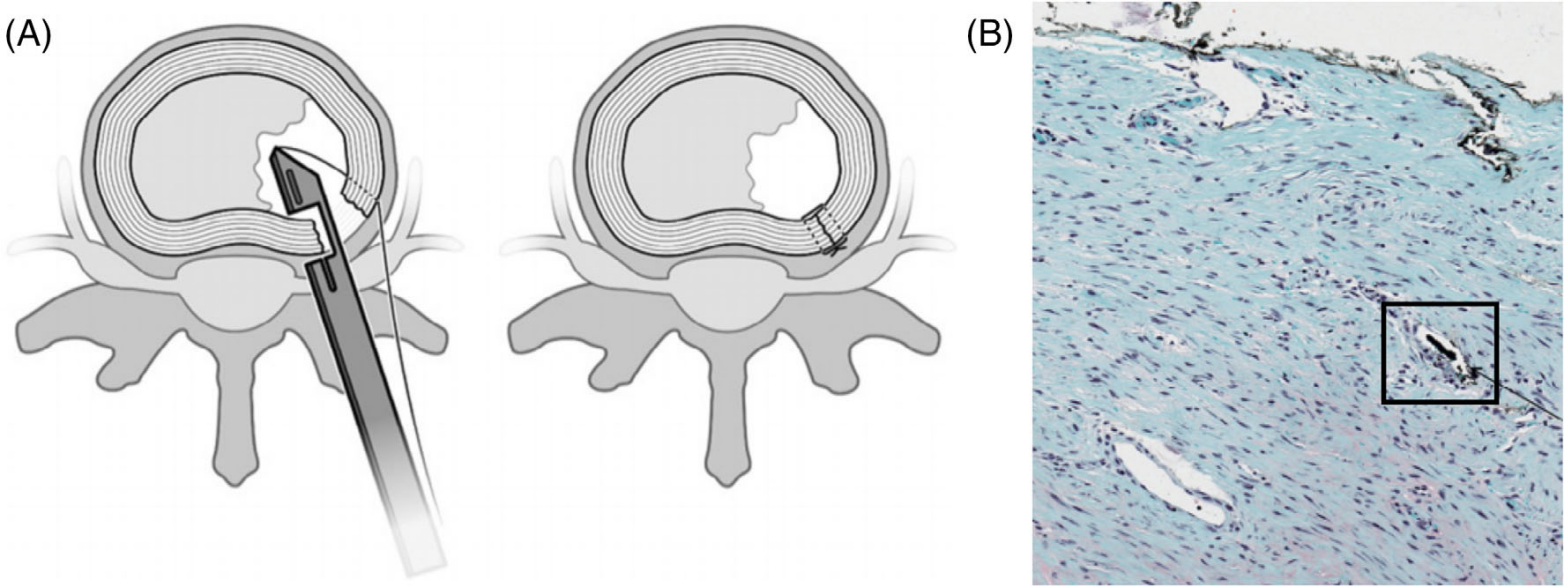
AnchorKnot Tissue Approximation Kit for AF repair. A, Graphic representation of the AnchorKnot suture system. Although intended as a minimally‐invasive system for the repair of AF lesions, the AnchorKnot Tissue Approximation Kit is only FDA approved for the visualization of the surgical field. B, Transverse histological AF specimen of a porcine cervical disc 4 weeks post‐repair using the AnchorKnot suture system (box) (scale bar not provided). Figures were republished with permission from The Spine Journal.82. AF, annulus fibrosus

**FIGURE 4:**
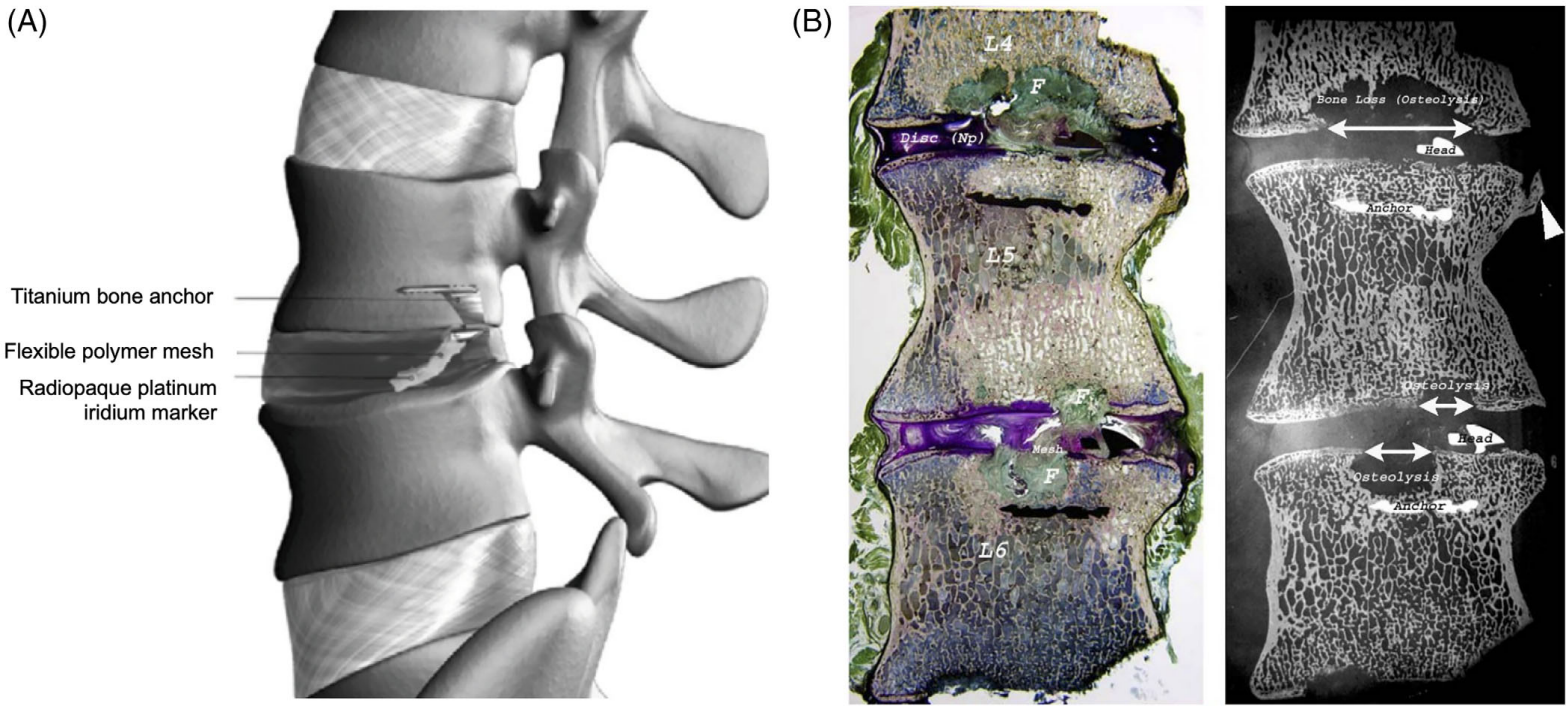
Barricaid annular closure device. A, Graphic representation of the Barricaid device with a flexible polymer mesh, designed to physically block the AF, a platinum‐iridium radiopaque marker to enable radiographic visualization, and a titanium alloy bone anchor that is implanted in the adjacent vertebral body for device fixation. Figure was republished with permission from the Journal of Pain Research.88, B, Undecalcified sagittal histologic section (left) stained with Villanueva's Osteochrome Bone Stain and corresponding microradiographic image (right) of baboon L4‐5 and L5‐6 lumbar spine levels 12 months postimplantation of the Barricaid device (scale bar not provided). “Anchor” and “head' label the device in the microradiographic image, which appears in black in the histological sample. Endplate disruption and osteolysis (white arrow) is apparent in both images, with replacement fibrotic tissue, F, apparent stained in green in the histologic sample. Osteophyte formation (white arrowhead) is highlighted in the microradiographic image. Figure was republished with permission from the Food and Drug Administration.83

Recently, a number of implantable devices and techniques have been developed to prevent re‐herniation, yet these systems do not biologically repair the AF. Examples of such systems include the AnchorKnot Tissue Approximation Kit (Anchor Orthopedics, Mississauga, ON, Canada) and Barricaid (Intrinsic Therapeutics, Inc., Woburn, Massachusetts). The AnchorKnot system enables minimally invasive visualization of the surgical field and is intended to minimize the removal of disc tissue and to close the AF defect with sutures (Figure 3A). Although reports indicate the device has been used in multiple clinics, systematic evaluation of its safety and efficacy for disc repair is not yet available, apart from an in vivo porcine study (Figure 3B).^80^ The device is currently only indicated for visualization of the surgical field. In contrast, Barricaid obtained FDA approval in 2019 for the prevention of disc re‐herniation following a limited discectomy (4‐6 mm tall and 6‐12 mm wide lesion) in the lumbar spine. The device has a titanium body that is inserted into the adjacent vertebra and a polyester fabric mesh that is placed adjacent to the disc lesion following discectomy to prevent recurrent herniation (Figure 4A). Several risks were identified following the long‐term implantation of the Barricaid device in a worst‐case baboon animal model study used to assess device safety.^81^ The study, reported in the summary of safety and effectiveness data FDA report, included implantation of the device at the L4‐L5 and L5‐L6 lumbar spine levels in nine mature male baboons. Evidence of vertebral endplate disruption, device subsidence beyond the endplates, inflammation, fibrosis, osteolysis, and osteophyte formation was found after 12‐months of device implantation, suggesting there were multiple risks associated with the Barricaid device implantation (Figure 4B).^81^ Since its FDA approval, early follow‐up clinical studies have reported beneficial outcomes 2 years postimplantation, such as the reduction in symptomatic disc re‐herniations and low complication rates; however, these reports also highlighted that device implantation led to higher prevalence of endplate changes.^82^, ^83^, ^84^, ^85^ The long‐term efficacy and safety of the device, especially concerning the damage of the vertebral bone and endplate during device fixation, remains to be determined.

## 5 ENDOGENOUS AF REPAIR

Although the root cause of annular tears varies, studies indicate that the endogenous repair process is common among all annular lesions. Animal models have enabled the investigation of events that occur after AF injury and the identification of some of the key cellular and molecular players involved in this process. Upon injury, acute inflammation arises near the lesion, characterized by an increase in the production of inflammatory molecules and the recruitment of inflammatory cells, such as macrophages and T lymphocytes.^86^, ^87^, ^88^, ^89^ A study in pigs in which herniations were induced via full thickness anterolateral circumferential AF tears showed that macrophages were more commonly present at the injury site than T cells. Notably, the tissue adjacent to the injury and control uninjured tissues showed no inflammatory cells.^86^ The rapid appearance of macrophages is likely related to their role in the clearance of damaged tissue and cellular debris. Increased macrophage presence was also detected in human disc herniations, where, in one third of the herniated samples, interleukin 1 beta (IL‐1β) expressing cells were also observed. This is characteristic of activated macrophages that increase the release of inflammatory cytokines once exposed to inflammatory stimuli.^88^, ^90^ An increase in IL‐1β was also noted in a rabbit annular stab model and a rat tail stab model.^91^, ^92^, ^93^ In the latter study, a triple stab injury increased IL‐1β, tumor necrosis alpha (TNFα), and IL‐8, with the inflammatory response lasting from 4 days for a single stab to 28 days for repetitive injury.^91^, ^92^, ^93^ This indicates that repetitive damage could potentially prolong the inflammatory response and promote tissue degeneration through increased catabolic activity.

In adult tissues, injury often results in cell death at the damaged site, creating regions that are nearly devoid of cells. For example, in a mouse IVD injury model, acute compressive overload resulted in a decrease in annular cellularity at the injury site that did not recover over 3 months postinjury.^94^ The cell death at the AF injury site, coupled with the low cellularity, dense ECM, and avascular nature of the tissue, likely impedes effective restoration of the tissue structure. This leads to a requirement for cellular migration and proliferation of surrounding cells in order to effectively re‐establish tissue composition and organization.

**FIGURE 5:**
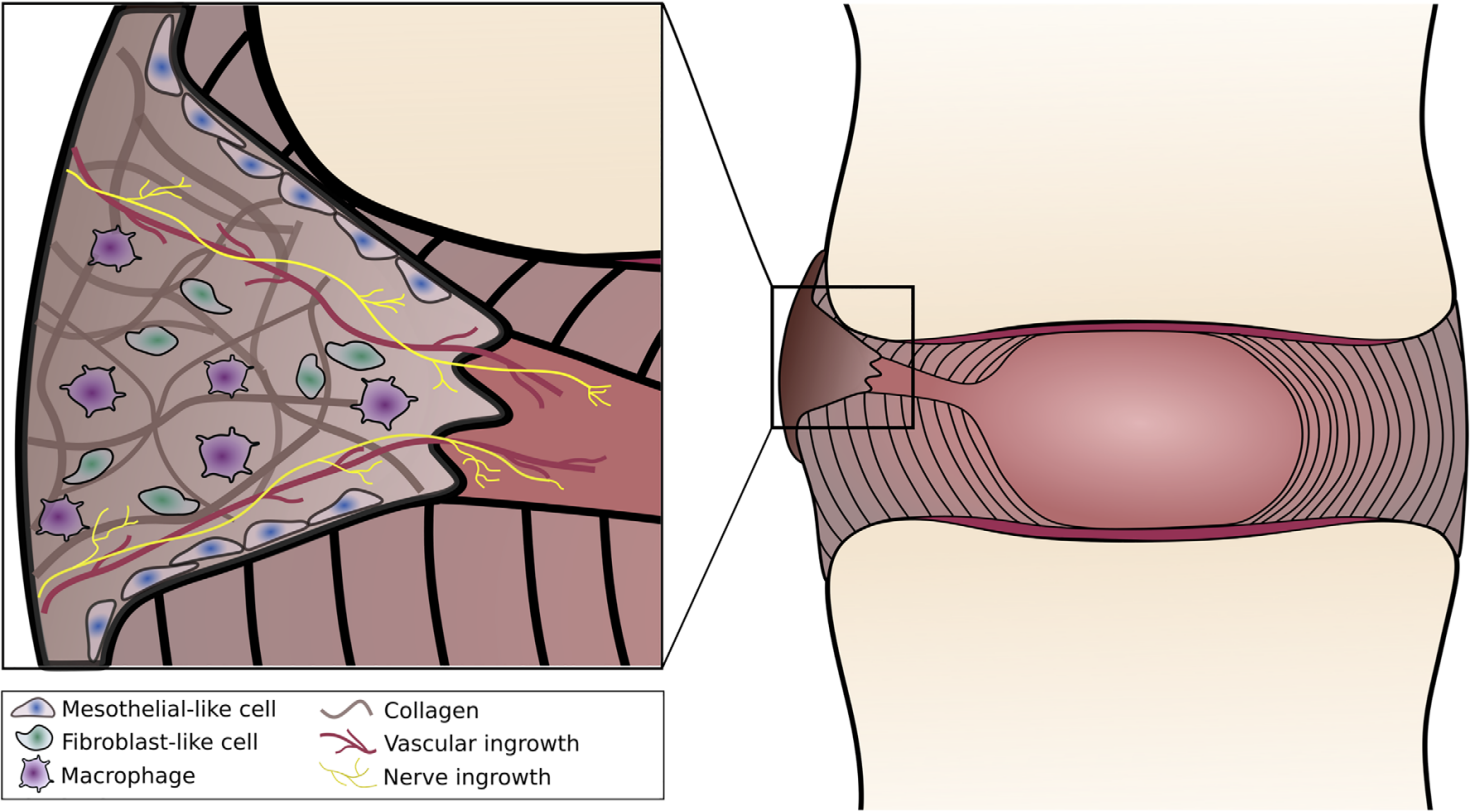
Schematic of endogenous scar tissue formation after AF injury. Scar tissue, originating from the periphery of the IVD, infiltrates into AF lesions, filling the outer third of the tear. This scar tissue is characterized by disorganized fibrous tissue, accompanied by vascular and nerve ingrowth. Macrophages and fibroblasts reside in the scar tissue, along with a mesothelial cell lining that forms along the edge of the native AF. AF, annulus fibrosus; IVD, Intervertebral disc

The unsuccessful healing of full‐thickness AF tears has been extensively described in human specimens and animal AF injury models. Most commonly, AF tears are infiltrated by fibrous tissue that originates from the periphery of the lesion (Figure 5). In most instances, full thickness defects are only partially closed, with only the outer third of the AF bridged by scar tissue and the remaining inner lamellae remaining open.^88^, ^95^, ^96^, ^97^ The peripheral source of this fibrous repair tissue suggests that extrinsic cell populations infiltrate the AF cleft and deposit the collagenous granulation tissue. In multiple studies, a single layer of plump fibroblast‐like cells has been observed in the AF lesion, lining the native tissue cleft in which the scar tissue infiltrates.^95^, ^98^ In a study that recreated full thickness box annulotomies and compared AF tissue healing to a full thickness stab wound in dogs, cells described as flat mesothelial‐type cells were found lining the sides of the stab injury tract at 12 weeks postinjury.^95^ The stab wound tract remained open through the AF thickness, except for a small fibrous cap at the periphery of the injury tract. Conversely, the box annulotomy injuries demonstrated a robust infiltration with a mature fibrous plug composed primarily of fibroblasts and collagen, with some areas of cartilaginous matrix in some specimens.^95^ The stab injury, which approximates full thickness radial AF tears, demonstrated very limited healing around the periphery of the injury tract. The lack of repair tissue suggests that the deposited scar tissue may be unable to resist the high intradiscal pressures that arise during axial loading, which may lead to the subsequent extrusion of NP material.

**FIGURE 6:**
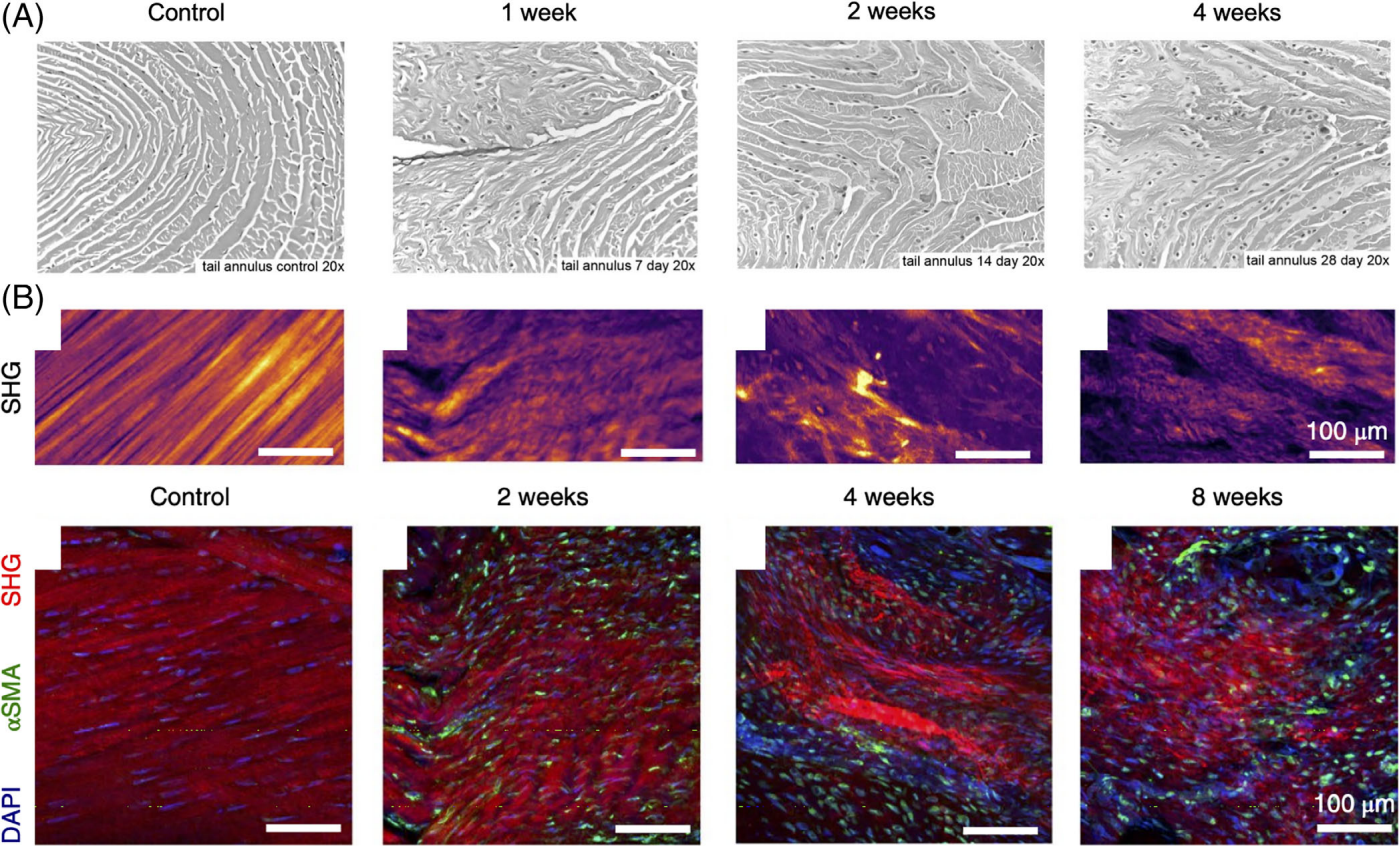
Endogenous AF structure and cell phenotype is altered following injury. A, Rat caudal lumbar disc AF lamella postpuncture of the IVD. Architectural and cellular changes are apparent over time, characterized by matrix disorganization and changes in cellular shape from stellar to round. Figure was republished with permission from Spine.95 B, Changes in AF collagen organization and cellular αSMA expression in the AF of punctured New Zealand white rabbit IVDs. (Top) SHG imaging of the outer AF demonstrated increasing fiber disorganization with time postinjury. (Bottom) This was accompanied by an increase in αSMA‐positive cells in the outer AF, indicating the emergence of a pro‐fibrotic response after injury. Figure was republished with permission from Nature Biomedical Engineering.102. AF, annulus fibrosus; IVD, Intervertebral disc; αSMA, alpha‐smooth muscle actin; SHG, second harmonic generation

Another factor that may limit endogenous healing of the AF are the drastic changes in cellular phenotype and homeostasis induced by the AF tear. One unique characteristic of the AF is the highly organized collagenous microenvironment that cells sense that is constantly under tension. When an AF tear occurs, the tension of the AF lamellar layers is lost which leads to lamellar buckling. Over time, the matrix becomes disorganized and important matrix proteins are lost, drastically changing the cellular microenvironment in which AF cells normally reside.^76^, ^92^ In a rat caudal and lumbar disc stab injury model, histologic evaluation of annular tears demonstrated collagen layer disorganization as early as 7 days postinjury and evidence of cellular metaplasia of AF fibroblasts into rounded cells resembling a chondrocyte‐like cell shape 14 days postinjury (Figure 6A).^92^ In a different study in which disc needle puncture was performed in New Zealand White rabbits, second harmonic generation imaging revealed a progressive loss of fiber organization over time.^76^ This was accompanied by an increase in cells that stained positive for alpha‐smooth muscle actin (αSMA) as soon as 14 days postinjury, indicating an emergent profibrotic phenotype, that remained apparent for up to 56 days after injury (Figure 6B).^76^ To decouple the effects of fiber organization from the in vivo environment, an electrospun scaffold with nanostructural characteristics resembling those of the native AF collagen fibers was used as a culture substrate with or without the application of tension. Using this system, the investigators showed that with increasing disorganization of fibers and a lack of pre‐strain, AF cells increased αSMA expression and their apoptotic response, which occurred in a contractility‐dependent manner.^76^ In a separate report, increased contractility and αSMA expression was observed in a CD146‐positive population of mouse AF cells.^77^ Contractility of these cells increased with greater rounds of in vitro expansion and with the addition of transforming growth factor β1 (TGF‐β1).^77^ Immunohistochemistry revealed that these CD146‐positive cells localized to the outermost layer of the AF in mouse IVDs and co‐expressed SM22α.^77^ These highly contractile peripheral cells could, therefore, be one of the cell types that migrate into AF lesions postinjury and play a role in the deposition of scar tissue during endogenous repair.

Nerve and vascular ingrowth also commonly accompany fibrous tissue infiltration into annular lesions. One study analyzed radiating tears in the posterior AF and circumferential tears in discs from humans above 50 years of age.^99^ They found that 14% of radial tears and 39% of circumferential tears had neovascularization.^99^ This new vascular supply arose from the posterior blood supply of the disc. In a study in which peripheral AF tears (5 mm in width and depth) were made in sheep, there was increased vascularization that infiltrated into the mid‐annulus in 10 of the 12 injured discs one‐month postinjury.^98^ While vascularization reached the middle of the AF, the granulation tissue that formed was restricted to the outer third of the AF, and this pattern remained unchanged after 18 months.^98^ Osteophyte formation was not observed after 1 month, yet by 18 months postinjury, osteophytes had formed in all treated discs.^98^ Importantly, discogenic pain has been postulated to be a consequence of nerve ingrowth along the vessels within the reparative tissue.^100^ The presence of nerves accompanying neovascular ingrowth has previously been reported, where nociception was confirmed via staining for calcitonin gene receptor protein, tyrosine hydroxylase, protein gene product (PGP) 9.5, and substance P.^99^, ^101^, ^102^ Small nerves and single axons with arborizing nerve endings formed in the outer scar tissue of rim lesions in sheep IVDs, penetrating an average of 3 lamellae (with a range of 1‐7 lamellae in depth).^99^ These nociceptive fibers were found at a greater density in the repair granulation tissue than in the adjacent AF.^99^ The ingrowth of nerves is attributed to the presence of inflammatory cytokines such as TNFα and interleukins.^103^ While neovascularization in the repair site may enhance the healing of the damaged tissue, the vessel growth that extends into the mid‐annulus should recede over time and localize to the IVD periphery, as seen in the adult healthy tissue. Similarly, the ingrowth of nerves into the fibrous reparative tissue is problematic if it penetrates deep into the IVD, as it may cause pain and discomfort upon tissue loading.^62^, ^99^, ^100^, ^101^, ^102^, ^103^, ^104^

The endogenous repair capacity of the adult AF is limited and does not restore native tissue structure and composition. The flaws in the process that impede the effective healing in the adult AF remain poorly understood. Recently, regenerative processes have been investigated using neonatal animal injury models, given the superior healing capacity of tissues at this stage of development.^48^, ^49^, ^105^, ^106^, ^107^ Mouse models also enable lineage tracing of cells. In a study comparing the repair of AF needle puncture injuries in Scleraxis green fluorescent protein (ScxGFP) neonatal and adult mice, neonates demonstrated accelerated AF healing and restoration of IVD biomechanical function. Conversely, adult discs did not recover after injury.^49^ While neonatal AF repair sites were fully occupied by collagenous repair matrix, the deposited scar matrix was not aligned; the adjacent native AF, however, remained highly organized and did not show degenerative changes 56 days postinjury.^49^ In contrast, adult injury lesions were filled by fibrous tissue in the outer portion and there was a marked loss of organization in the adjacent AF.^49^ Interestingly, ScxGFP‐negative cells or cells not identified as tenocytes or AF cells, were found in the injury site 1 month postherniation in adult injuries. This suggests either the involvement of extrinsic cells or the loss of Scx expression in cells participating in the repair process. To characterize the cellular dynamics and identify the cellular players partaking in neonate and adult AF healing, cell apoptosis, proliferation, and lineage tracing were also investigated.^48^ Neonatal AF regeneration was found to be mediated by the proliferation and recruitment of Scx‐lineage cells that ultimately lost Scx expression, adopted a stem cell‐like phenotype, proliferated, and finally, reacquired Scx expression 56 days postinjury.^48^ Approximately 53% of the injury tract cells in the neonate had restored Scx expression while only ~7% of cells in the adult showed expression of this marker.^48^ While nonScx‐lineage cells such as sonic hedgehog‐lineage cells (NP cells), macrophages, and myofibroblasts were transiently present in the neonate AF lesion, pluripotent stem cells, endothelial cells, and pericytes were not detected.^48^ Interestingly, although the adult AF injury site was largely devoid of cells, a majority (~90%) of the cells that were present were not of the Scx‐lineage.^48^ This suggests that Scx‐lineage cells may be required for accelerated adult AF repair and effective restoration of IVD mechanical function.

To enhance the reparative processes of the adult AF, an improved understanding of the limitations of the endogenous repair is required to determine whether AF regeneration occurs through the differentiation of recruited and/or resident progenitor cells, the replication of differentiated cells or the transdifferentiation of resident cells. This fundamental knowledge would enable the design of exogenous delivery systems that are tailored to the cellular mechanisms involved.^15^ Although transdifferentiation of recruited cells has been implicated in fetal AF repair,^48^ the mechanisms involved in the repair of the adult tissue remain less understood. This is furthered by the negative effects of aging on vital cellular processes, such as proliferation and differentiation.^108^, ^109^ In the adult AF, healing is characterized by the infiltration of fibrous scar tissue accompanied by vessel and nerve ingrowth at the IVD periphery. Preventing the infiltration of these exogenous cells may potentially deter the formation of the fibrous cap and the extensive infiltration of vessels and nociceptive nerves. The re‐establishment of the tissue composition and organization would therefore rely on resident AF cells; however, the dense ECM, the low cellularity, and avascular nature of the adult tissue make this a challenging venture.

Methods that deliver specialized cells or that increase the recruitment of endogenous cells to the site of injury could enable a faster functional recovery of tissue structure.^15^, ^16^, ^17^ The additional provision of physical and soluble cues may provide a template for the establishment of cellular organization and guide cellular biosynthesis to ultimately, recover tissue structure and function. Many groups have employed biomaterial‐based approaches for AF repair that provide guidance signals at the site of injury.^8^, ^17^ This area of research is summarized in recent reviews by our group and others covering biomaterials used for AF repair, cellular delivery and recruitment approaches, and biofactor delivery to enhance tissue healing.^15^, ^16^, ^17^, ^109^, ^110^, ^111^, ^112^, ^113^

Repair methods should also consider the inflammatory environment. The involvement of macrophages after tissue injury is vital for early wound healing. Initially, macrophages guide the innate immune response by phagocytosing cellular debris and releasing agents that promote anti‐microbial activity; over time, the cells switch to a pro‐regenerative phenotype and produce molecules that drive cellular proliferation and tissue repair.^94^ Persistent inflammation can decrease wound healing and increase fibroblastic activity and scarring, ultimately hindering organ function.^114^, ^115^ A dysregulated inflammatory response can also increase the catabolic degradation of the native AF ECM. This seems to be the case for the adult AF where the native AF tissue loses its organization along the periphery of the injured tissue during healing, while native tissue organization is maintained during fetal AF repair.^48^, ^49^ Reprogramming the immune environment while harnessing cellular function could help to effectively restore IVD mechanical function and re‐establish the highly specialized tissue composition and organization to prevent recurrent IVD herniations.

## 6 FUTURE DIRECTIONS

It is clear that the mechanical failure of the AF is central to the pathophysiology of disc herniations. Symptomatic IVD herniations are surgically treated via microdiscectomy to relieve patient symptoms yet the AF, which relies on its structure and architecture to achieve its mechanical function, is left unrepaired. Currently, there is only one device that is FDA‐approved to prevent recurrent herniation postmicrodiscectomy. Although the device has been shown to decrease the rate of re‐herniation for up to 2 years postrepair, the long‐term effects of device implantation including stability, wearing, and adjacent tissue health have yet to be reported. The Barricaid device acts as a barrier against the AF lesion that gave way to the herniation; however, the device does not repair the AF, compromising the mechanical integrity and function of the IVD load‐bearing unit. A strategy that biologically repairs and restores the AF to native, or near‐native properties, may be necessary. Similarly, the recapitulation of native AF biomechanics will likely mitigate the degenerative cascade of the motion segment including the NP and the posterior elements (eg, facet joints). As lumbar disc herniations present in a relatively younger patient population, and recurrent disc herniations are associated with surgical lumbar fusions,^116^ restoration of spinal motion segment mechanics may obviate progressive degenerative disease and the associated incidence of re‐herniation, fusion, and adjacent segment disease.

The ideal AF repair device should demonstrate safety and efficacy, providing superior outcomes when compared to microdiscectomy intervention. The benefits for the implantation of such repair device should outweigh the risks associated with the surgical procedure. Ultimately, an effective implant should decrease the risk for re‐herniation by sealing the AF lesion and engaging the endogenous healing response to recapitulate the complex native tissue structure, organization, and function. By maximizing the involvement of pro‐regenerative, anabolic cells at the repair site and preventing chronic inflammation, a robust integration between the repair device and the native tissue may be achieved.^15^ The complex anatomy of the IVD requires devices to enable minimally‐invasive implantation, demanding the implant to have a small footprint while providing robust mechanical stability and anchorage to the repair site. Due to the patient‐to‐patient variations in herniation location and severity, repair systems should also be customizable for each patient to seamlessly integrate with the injured disc. Approaches that minimize AF tissue resection and further damage to the IVD during implantation will minimize the risk for inferior healing outcomes and for further degeneration of the tissue. Furthermore, damage to adjacent tissues (eg, vertebral bodies, nerve roots, muscle, ligaments, etc.) should be kept to a minimum to prevent chronic inflammation, the degeneration of the spinal unit, and the formation of osteophytes. After implantation, the device should provide long‐term prevention of re‐herniation while enduring dynamic complex loads without wearing or catastrophically failing. These structural, mechanical, biological, and functional requirements may serve as guiding principles for the design of future devices that repair the AF postdisc herniation.

## CONFLICT OF INTEREST

Ana P. Peredo has no competing financial interests.

Sarah E. Gullbrand has no competing financial interests.

Harvey E. Smith has no competing financial interests.

Robert L. Mauck has no competing financial interests.

## AUTHOR CONTRIBUTIONS

All authors have contributed to the drafting of the manuscript and its critical revision, and have approved the final version.
